# Are Lay People Good at Recognising the Symptoms of Schizophrenia?

**DOI:** 10.1371/journal.pone.0052913

**Published:** 2013-01-02

**Authors:** Philip Erritty, Taeko N. Wydell

**Affiliations:** Brunel University, Middlesex, United Kingdom; University of Cardiff, United Kingdom

## Abstract

**Aim:**

The aim of this study was to explore the general public’s perception of schizophrenia symptoms and the need to seek-help for symptoms. The recognition (or ‘labelling’) of schizophrenia symptoms, help-seeking behaviours and public awareness of schizophrenia have been suggested as potentially important factors relating to untreated psychosis.

**Method:**

Participants were asked to rate to what extent they believe vignettes describing classic symptoms (positive and negative) of schizophrenia indicate mental illness. They were also asked if the individuals depicted in the vignettes required help or treatment and asked to suggest what kind of help or treatment.

**Results:**

Only three positive symptoms (i.e., Hallucinatory behaviour, Unusual thought content and Suspiciousness) of schizophrenia were reasonably well perceived (above 70%) as indicating mental illness more than the other positive or negative symptoms. Even when the participants recognised that the symptoms indicated mental illness, not everyone recommended professional help.

**Conclusion:**

There may be a need to improve public awareness of schizophrenia and psychosis symptoms, particularly regarding an awareness of the importance of early intervention for psychosis.

## Introduction

Schizophrenia is recognised as a particularly disabling psychiatric disorder for sufferers and their families, and often carries a poor long-term outcome [1], [2]. The prevalence in the population is generally estimated between 0.5–1.0% [3]. Early detection and the initiation of effective treatment, particularly for people suffering from a first-episode of schizophrenia, is becoming an increasingly important issue in mental healthcare [4]. The duration of untreated psychosis (DUP) has been widely recognised in recent years as a potentially important predictor of illness outcome in schizophrenia [5]. DUP is understood as the period of time from the development of the first symptoms of psychosis to the commencement of appropriate intervention measures, such as anti-psychotic drug treatment [6]. The issue has raised the importance of early detection and intervention for people developing psychotic illness [7]. However, Lieberman [8] rightly points out that more research into the reasons for the delays in effective treatment for psychosis is required. It has been suggested that factors such as poor public awareness and a failure to recognize the symptoms of psychosis may play an important role in delaying the initiation of treatment for psychosis [9], [10]. The current study explores lay perceptions of schizophrenic symptoms and the need to seek help for symptoms in the general population. This study also aims to bridge-the-gap between clinical research on schizophrenia and research on how schizophrenia is thought about in the general population, given that few studies have attempted to combine these two important research areas.

### Symptom Detection and Help-seeking in Psychosis

In a recent study, O’Callaghan et al. [9] found that the duration of untreated psychosis could last for almost three years. With respect to this sobering reality, a critical question for social psychiatry might be why there are such delays in the initiation of treatment for psychosis. Indeed, O’Callaghan et al. reflect that understanding the causes of such long treatment delays for psychosis may be a particularly difficult task because numerous factors play a role, including inadequate public awareness of the symptoms, difficulty for the individual to detect more subtle symptoms and health professionals to differentiate diagnostically, combined with the lack of patient insight.

Along with these factors related to symptom detection and recognition, Singh and Grange [11] highlight the complex nature of help-seeking and the pathways to care for individuals suffering from psychosis in that they can be highly variable, and can involve social services, police authorities and emergency services. It is an unfortunate reality that individuals suffering from psychosis are more likely to come into contact with the police and criminal justice system than people not suffering from mental illness, and that this is likely due to unusual behaviour as a result of their illness [12], [13]. Rogler and Cortes [14] referred to help-seeking and pathways to care as the contact made by individuals suffering from mental illness and their families with individuals or organisations in an effort to seek help, as well as help that is given in response to such efforts. In a meta-analysis study of research into pathways to care and help-seeking for psychosis, Singh and Grange [11] concluded that whilst the pathways to care for people exhibiting a first-episode psychosis could be highly variable, they found that much research reports health professionals as the first help-seeking point of contact (see Singh and Grange [11] for full review).

However, these researchers also highlighted certain factors that may affect pathways to care and help-seeking for psychosis, such as ethnicity [15], the role of families and carers [10], and under-recognition of prodromal (i.e., pre-psychotic) and early psychotic symptoms [16].

It is the recognition of the symptoms of schizophrenia that is of particular interest to the current study presented here. Prodromal symptoms are believed to be reflected by a number of non-specific and variable changes in the behaviour of an individual during the early stages of schizophrenic illness [17], [18], [19]. These changes of behaviour often include attenuated positive symptoms such as unusual thought content, suspiciousness, hallucinatory behaviour and conceptual disorganization as well as negative symptoms such as depressed mood, low energy, lack of motivation (apathy), social withdrawal and affective flattening [20] They may also manifest themselves via a deterioration or alteration in functioning at work or in school, or through illicit drug use [17], [18], [19].

As was mentioned previously, there has been a growing movement for early intervention in the prodromal phase of illness in order to prevent the damaging effect of a first-episode psychosis [7], [17], [21]. With regard to the findings of under-recognition of prodromal and early psychotic symptoms affecting help-seeking, the research conducted by Addington et al. [16] is of particular interest. They investigated factors that may be influencing delays in treatment and adequate pathways to care, in particular, the types of symptom presentations that caused concern to, and influenced, individuals or their families to seek help. Using a sample of first-episode psychotic patients, they found that it was the presence of psychotic symptoms that caused most concern and most influenced help-seeking and achieving successful pathways to care.

O’Callaghan et al. [9] found that only 40% of their first-episode psychotic patient sample sought help even after the onset of florid psychotic symptoms, and that even fewer sought help during the prodromal phase. Perkins et al. [22] also suggested that an underestimation of the importance of symptoms and help-seeking may delay the attainment of effective treatment. Lincoln et al. [10] have questioned community and public knowledge of the importance of early intervention, and Lieberman [8], referring to the dangers of a long duration of untreated psychosis, questioned how well individuals developing psychosis recognize the symptoms as indicating mental illness. Furthermore, having identified a low level of help-seeking by psychosis sufferers during the early stages of illness, Kohn et al. [23] suggest that public awareness campaigns are needed to help shorten the period between first symptoms and help-seeking behaviour. In this sense, much research is indicating a need for greater ‘mental health literacy’ in the general population.

### Mental Health Literacy

The term ‘mental health literacy’ was defined by Jorm et al. [24] as ‘knowledge and beliefs about mental disorders which aid their recognition, management or prevention’. Key components of this concept include lay people’s ability to recognise specific disorders or types of distress and their knowledge and beliefs about appropriate help-seeking. Jorm [25] further identified that the symptom-management of people experiencing psychological distress (or witnessing it in close contacts) will be influenced by their mental health literacy. Jorm [25] suggested that an increase in mental health literacy in the population would help develop preventative measures and assist in early intervention for psychiatric illnesses.

### Research into Lay Perceptions of Schizophrenia

Because schizophrenia is a vastly complex disorder, and its multifarious components mostly concern mental health professionals and clinical researchers, there appears to be a resulting gap in the understanding of perceptions of schizophrenia in lay populations, and few studies have been conducted [26]. A study conducted by Luty et al. [27] investigated the British public’s understanding of the term ‘schizophrenia’, however, participants were only asked to provide a short response to a single question, “What do you understand by the term ‘schizophrenia’?”. They found that 42% of respondents mentioned at least one first-rank symptom including auditory or visual hallucinations, thought disorder, delusions or passivity experiences. Lauber et al. [26] conducted a more comprehensive study of lay perceptions of mental illness, which included schizophrenia and depression vignettes. They found that the schizophrenia vignette, depicting a person with “schizophrenia fulfilling the respective DSM-III-R criteria” (p. 249) was better recognized as mental illness than the depression vignette. However, there was little depth regarding specific schizophrenia symptoms. Lauber et al., concluded that poor public knowledge of mental disorders (or ‘mental health literacy’) was wide spread.

There have been more in-depth studies of lay perceptions specific to schizophrenia, including Pote and Orrell [28], and Stone and Finlay [29], who investigated cultural differences in the perceptions of schizophrenia. For example, Pote and Orrell asked 190 participants aged above 18 from five ethnic groups (i.e., Afro-Caribbean, Bangladeshi, Indian, Sub-Saharan African, and White British) in London, UK, their perception of schizophrenia symptoms using the Perceptions of Mental Health Problems Questionnaire. They found that “ethnic group was the best predictor of differences that were found across schizophrenia symptoms” (p.18), and suggested that these differences should be considered when developing mental health services “to meet the needs of multi-cultural Britain” (p.18).

Similarly Stone and Finlay [29] asked 128 adult students aged over 16 of different ethnicities (i.e., Asian, Black/African-Caribbean, Chinese, Mixed and White/European) from further education colleges in London, UK to complete the Social Perceptions Questionnaire (SPQ). The SPQ comprises seven vignettes, four of which describe schizophrenia symptoms, and two of which describe “unusual non-symptom behaviours not classified as psychiatrically pathological” (p.248), and one which describes a diagnosis of schizophrenia. They found that African-Caribbean participants perceived the schizophrenia diagnosis and three of the four schizophrenia symptoms as less socially stigmatizing than While/European participants. In turn, White participants were more likely to suggest seeking help for symptoms including a health professional than African-Caribbean participants. In order to account for these cultural differences in relation to the willingness to receive medical treatment for schizophrenia, Stone and Finlay interestingly suggested “…Given that health services are not always seen as appropriate help for symptoms of schizophrenia by different ethnic groups, joining with other services – e.g. churches, leisure and voluntary projects – may provide opportunities for people to view health services as an equally valid and useful means of help…” (p. 257).

In the current study, the research methodologies developed by Pote and Orrell [28], and Stone and Finlay [29] are also employed, particularly regarding the concept of investigating schizophrenia symptom recognition (or labelling) and help-seeking beliefs in lay populations. The study also focused on schizophrenia perceptions and subsequent help-seeking. With reference to the important clinical issue of long durations of untreated psychosis (DUP) and recognition of Jorm’s [25] assertions that public mental health literacy may affect prevention and intervention, this study aimed to further explore and add more knowledge to our understanding of lay perceptions of the symptoms, and consequent help-seeking for schizophrenia.

### Hypotheses

#### 1. Schizophrenia symptom perceptions

Schizophrenia is typically characterized by having positive symptoms such as ‘hallucinatory behaviour’, ‘unusual thought content’, ‘suspiciousness’ and ‘conceptual disorganization’ (i.e.,disorganized thinking or behaviour), and also negative symptoms such as ‘avolition-apathy’(i.e., lack of motivation), ‘alogia’ (i.e. poverty of speech), anhedonia-asociality’ (i.e., lack of positive emotion and desire to form relationships) and ‘affective flattening’(i.e., inability to experience a normal range of emotion). It was therefore hypothesized that there would be differences in the perception of positive and negative symptoms of schizophrenia among lay-participants. In particular, it was hypothesized that positive symptoms would be more readily identified as indicating mental illness than negative symptoms. This hypothesis was based on the previous research pertinent to this question by Pote and Orrell [28].

#### 2. Help-seeking perceptions of schizophrenia symptoms

Due to the lack of research regarding this particular research question, and the exploratory nature of the study, 2-tailed hypotheses were put forward.

It was hypothesised that participants would either suggest help or treatment for the symptoms of schizophrenia (treatment beliefs) or not. This hypothesis was broadly based on Stone and Finlay’s [29] work described above.

#### 3. Treatment recommendations for schizophrenia symptoms

It was also hypothesised that participants would recommend professional help more than other help or don’t know for the symptoms of schizophrenia (treatment suggestions). This hypothesis was also broadly based on Stone and Finlay’s work.

## Method

### Participants

A total of 88 participants took part in the study. 45 were male (age range 18–67, mean age 33.2; SD = 15.81) and 43 were female (age range 18–65, mean age 33.8; SD = 14.88). A wide age range was represented in the samples. Half the participants were white-British students recruited on the campus grounds of Brunel University in Uxbridge (aged between 18 and 23), and the other half of the participants were white-British in ethnicity recruited in Lewes in Sussex, UK (where the majority of residents are generally considered as middle-class) (aged between 25 and 67). None of the participants had educational qualifications in psychology at either degree level or A-level. This was in order to help control for participants with any formal education in schizophrenia. The current study was approved by the Psychology Ethics Committee at Brunel University.

### Stimuli, Materials and Procedure

The participants were approached in-person with the questionnaire pack and guided through the informed consent form and the procedure for completing the questionnaire. The pack also contained a screening for the participants to indicate their age, gender and educational experience in psychology, and an additional instruction sheet for completing the questionnaire was attached for participants to reference. Participants were then provided with a debriefing form after completing the questionnaire.

As shown in Table 1, the questionnaire used in the current study contains eight schizophrenia symptom vignettes and two non-symptom filler vignettes from McEvoy, Schooler, Friedman, Steingard and Allen [30]. It should be noted that although symptom vignettes are an interesting and implicit method of investigating attitudes towards schizophrenia symptoms in general, it is undeniable that reading descriptive accounts of the positive and negative symptoms of schizophrenia is qualitatively different from actually experiencing the symptoms either as a patient or as a relative (and then acting, or not acting upon them). McEvoy et al. created these symptom vignettes using the Brief Psychiatric Rating Scale [31] and the Scale for the Assessment of Negative Symptoms [32] as their sources.

**Table 1:** Vignettes used in Questionnaire.

SYMPTOM VIGNETTES
**Avolition-apathy**
He does not have much energy. He sleeps a lot and, when up, is quite satisfied to sit around and not do much. He does little or nothing spontaneously, on his own initiative. He has to be asked or told to do things, even simple things like taking a bath or putting on clean clothes. Even if he begins a task, he is soon worn out and stops. It is tough to finish things.
**Alogia**
He does not have much energy. He sleeps a lot and, when up, is quite satisfied to sit around and not do much. He does little or nothing spontaneously, on his own initiative. He has to be asked or told to do things, even simple things like taking a bath or putting on clean clothes. Even if he begins a task, he is soon worn out and stops. It is tough to finish things.
**Anhedonia-asociality**
He does not find much interesting. Things that had previously been attractive or stimulating just do not seem to matter anymore. He does not get out with others much. It just does not seem worth the effort and trouble. Not much is fun. Not much is exciting. It is simpler just to stay at home and take things easy by himself.
**Affective Flattening**
He seems to be affected less by things, to show less emotion. He laughs less, cries less, worries less. It is a quiet state, a bit dull. Things just do not seem to affect him like they used to. He seems to have fewer feelings, and the feelings are not as strong. Even his face shows less expression.
**Hallucinatory behaviour**
He began to notice people talking about him. At first he was not sure who it was talking or why they talked about him. People talked about him in many different places and he gradually became used to it. Sometimes at night they would be outside his window or in the next apartment. Sometimes it was almost like telepathy. Sometimes they said very nasty things.
**Suspiciousness**
It became very clear to him that something was definitely going on. They had singled him out and they meant to cause him trouble. Some very powerful people intended to harm him, and these people left clues everywhere in order to threaten and worry him. He had to be very cautious because these people seemed to know an incredible amount about him. Perhaps they were secretly monitoring him.
**Unusual thought content**
He had some very surprising experiences. People seemed able to know about his thoughts. He would just think about a topic and, next thing, they would broadcast that very topic over the radio or TV. People on the street would signal that they knew what he was thinking. Sometimes signals appeared in things he was reading that showed how much they knew about him. Sometimes these people would put their thoughts into his mind. That felt strange.
**Conceptual disorganization**
It was hard for him to maintain his concentration. Sometimes lots of things would crowd into his mind in a big jumble, and he would lose his train of thought. Noises or lights would easily distract him. Sometimes he realized how many things were connected in so many ways and that became confusing – other people could not see some of the connections. Sometimes when a lot was going on it was simply too much and his thoughts would just stop for a while; he would go into a daze.
**NON-SYMPTOM FILLER VIGNETTES**
**Extroversion**
He is always the first person to speak in a group. Sometimes he can talk for 10 minutes without giving others a chance to say their point of view. When people do interrupt he will often make a critical comment, which silences them. He does things spontaneously, in reaction to what is going on around him, and may not think them through beforehand.
**Affectionate**
He is very affectionate, and likes most people he meets. On meeting a friend or someone he has only met on a couple of occasions he will hug them and kiss them on the cheek. He will talk to others in a very loving manner, and always show his feelings. He is often expressive with his hands when talking and will touch others frequently.

The symptom vignettes contained four positive symptoms of schizophrenia, which include ‘suspiciousness’, ‘hallucinatory behaviour’, ‘unusual thought content’ and ‘conceptual disorganization’, and four negative symptoms of schizophrenia, which include ‘avolition-apathy’, ‘anhedonia-asociality’, ‘alogia’ and ‘affective flattening’. Pote and Orrell [28] and Stone and Finlay [29] subsequently used the vignettes in general population samples. The two non-symptom filler vignette (‘extroversion’ and ‘affectionate’) were taken from Stone and Finlay [29] and have been used in this study as distracters in order to help balance responses. A statement was provided at the start of the questionnaire, which read ‘The behaviours and experiences described in the vignette implies mental illness’. Participants were told to assume that the behaviours had been going on for at least two weeks [28].

A question was then provided below the statement asking, ‘Do you agree or disagree with this statement?’ Participants were then asked to indicate their responses per vignette on a five-point Likert scale (1 = ‘Strongly agree’, 2 = ‘Somewhat agree’, 3 = ‘Not sure’, 4 = ‘Somewhat disagree’, 5 = ‘Strongly disagree’). *It is important to note that participants were not made aware that any of the vignettes described schizophrenia symptoms.*

- In order to assess treatment beliefs for each vignette participants were asked ‘Does this indicate any need for help or treatment?’ and would then indicate either ‘Yes’ or ‘No’.
- In order to assess treatment suggestions, participants were then asked, ‘if so, what kind of help or treatment?’ in an open-ended question, replicating Stone and Finlay [29], from which participants could write their suggested help or treatment.

Participants took on average 10 minutes to complete all the questionnaires. No participation fee was paid to the participants.

## Results

### Perceptions of Positive and Negative Symptoms

The breakdown of participant responses per symptom vignette is shown in Table 2.

**Table 2:** Participant responses (frequency and percentages) per vignette across the ‘agree’, ‘not sure’ and ‘disagree’ response categories, with chi-square analyses.

N = 88	Agree	Not sure	Disagree	?_2_	*P*
Avolition-apathy	52(59.1%)	15(17.0%)	21(23.9%)	26.886	0.001*
Alogia	19(21.6%)	21(23.9%)	48(54.5%)	17.886	0.001*
Anhedonia-asociality	50(56.8%)	14(15.9%)	24(27.3%)	23.545	0.001*
Affective flattening	38(43.2%)	27(30.7%)	23(26.1%)	4.114	0.128
Hallucinatory behaviour	77(87.5%)	10(11.4%)	1(1.1%)	117.568	0.001*
Unusual thought content	66(75.0%)	17(19.3%)	5(5.7%)	71.205	0.001*
Suspiciousness	63(71.6%)	21(23.9%)	4(4.5%)	62.886	0.001*
Conceptual disorganization	49(55.7%)	17(19.3%)	22(25.0%)	20.205	0.001*

* *P<0.001*.

The ‘Agree’ category was comprised of ‘strongly agree’ and ‘somewhat agree’ Likert responses (values 1 and 2), and the ‘Disagree’ category was comprised of ‘strongly disagree’ and ‘somewhat disagree’ Likert responses (values 4 and 5). As can be seen from Table 2, on average, the participants tended to perceive positive symptoms as more indicative of mental illness than negative symptoms.

A series of Chi-square tests were conducted to see the association between the three different response types and a given symptom vignette. The results (also shown in Table 2) revealed that all the vignettes were significant, all at p<.0001 except for ‘Affective Flattening’ (p>.05). Post-hoc analyses revealed that in terms of the *negative symptoms,* participants were more likely to agree that ‘Avolition-apathy’ (χ_2_ = 26.89, p<0.001) and ‘Anhedonia-asociality’ (χ_2_ = 23.55, p<0.001) were the vignettes indicated mental illness. In contrast, for ‘Alogia’, participants were more likely to ‘Disagree’ that the vignette indicated mental illness than ‘Agree’ (χ_2_ **= **12.56, p<0.001) and than ‘Not sure’ (χ_2_ **= **10.57, p<0.005).

In terms of the *positive symptoms*, for ‘Hallucinatory behaviour’ participants were more likely to ‘Agree’ than ‘Disagree’ (χ_2_ **= **74.05, p<0.001) and to rate as ‘not sure’ (χ_2_ = 51.60, p<0.001) that the vignette indicated mental illness. Further, participants were also more likely to rate as ‘not sure’ than to disagree (χ_2_ = 7.36, p<0.01). A very similar pattern was found for ‘Unusual thought content’ (χ_2_ **= **52.41 p<0.001; χ_2_ **= **28.93 p<0.001; and χ_2_ **= **6.55 p<0.05 respectively) and ‘Suspiciousness’ (χ_2_ **= **10.27 p<0.001; χ_2_ **= **21.00 p<0.001; and χ_2_ **= **11.56 p<0.001, respectively). For ‘Conceptual disorganisation’ participants were also more likely to ‘agree’ that the vignettes indicated mental illness than to disagree (χ_2_ = 10.27, p<0.005) or to rate as ‘not sure’ (χ_2_ = 15.52, p<0.001), but were no more likely to disagree than to rate as ‘not sure’.

### Treatment Beliefs

The results of the Chi-square tests revealed that for most symptom vignettes the participants **who had originally agreed** that a vignette was indicative of mental illness then suggested that treatment/help was required, all at p<001. The only exception was for ‘Alogia’ (χ_2_ = 1.32, p = 0.25), where these participants were no more likely to recommend treatment/help than not.

**Figure 1:**
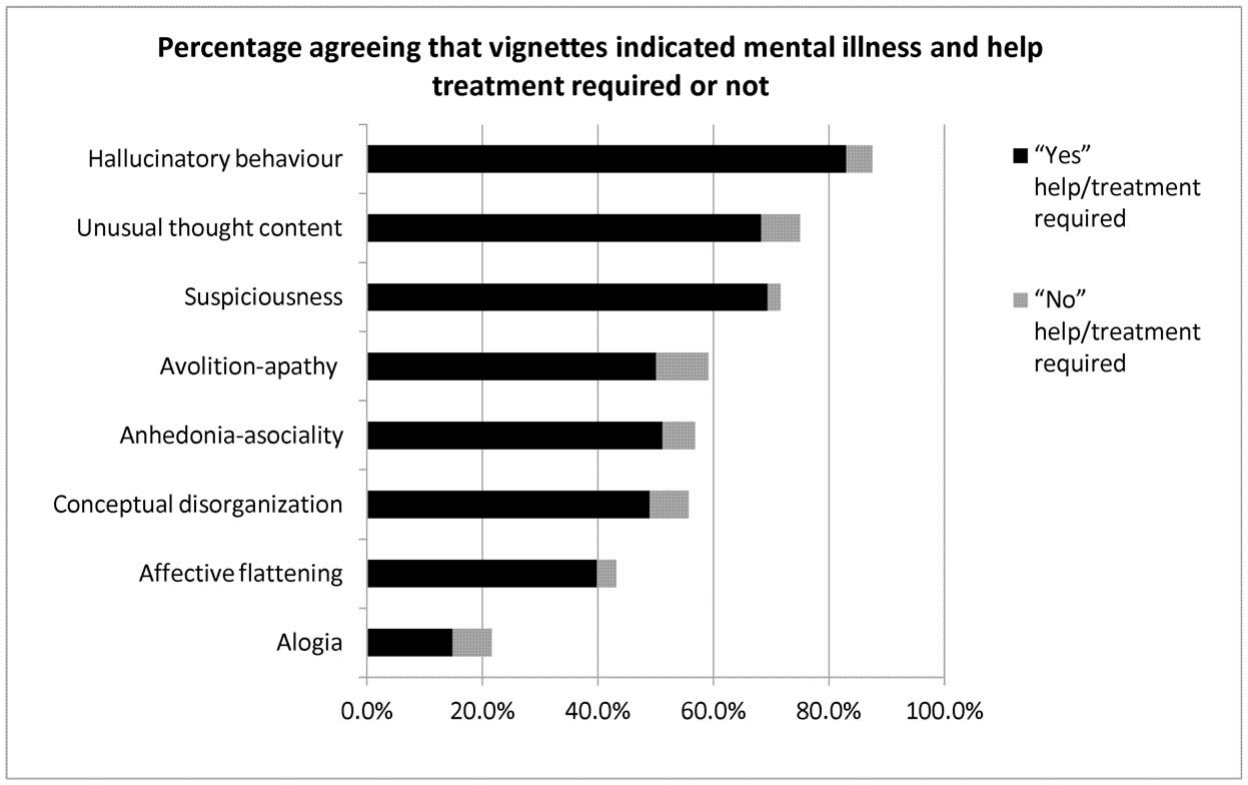
Treatment Belief - frequencies, percentages and chi-square analyses of the decisions on whether help is required or not.

The breakdown of the data for those who initially agreed that the vignette indicated mental illness and suggested “YES help/treatment required” or “No help/treatment required” for each symptom is illustrated in Figure 1.

As illustrated in Figure 1, for ‘Hallucinatory behaviour’, more than 80% of the current sample population who initially agreed that the vignette indicated mental illness suggested help/treatment required, and for ‘Unusual thought content’ and ‘Suspiciousness’, around 70% of them suggested help/treatment is required. These three symptoms are all *positive symptoms*.

For ‘Avolition-apathy’ (negative symptom), ‘Anhedonia-asociality’ (negative symptom) and Conceptual disorganisation (positive symptom), **only** around 50% of the current sample population suggested help/treatment is required. For ‘Alogia’ (negative symptom), less than 20% suggested help/treatment is required. On the whole the participants perceived the positive symptoms as more indicative of mental illness than negative symptoms.

### Treatment Suggestions

For this analysis, participants were asked, in an open-ended question, to suggest some form of help or treatment for each symptom vignette. The answers given were categorized into ‘professional help’ (i.e. psychiatric assessment, GP, etc.), ‘other help’ (i.e. family & friends, the police, etc.) and ‘don’t know’. This technique for categorization had been used by Stone and Finlay [29], and had been assessed for content inter-reliability.

**Figure 2:**
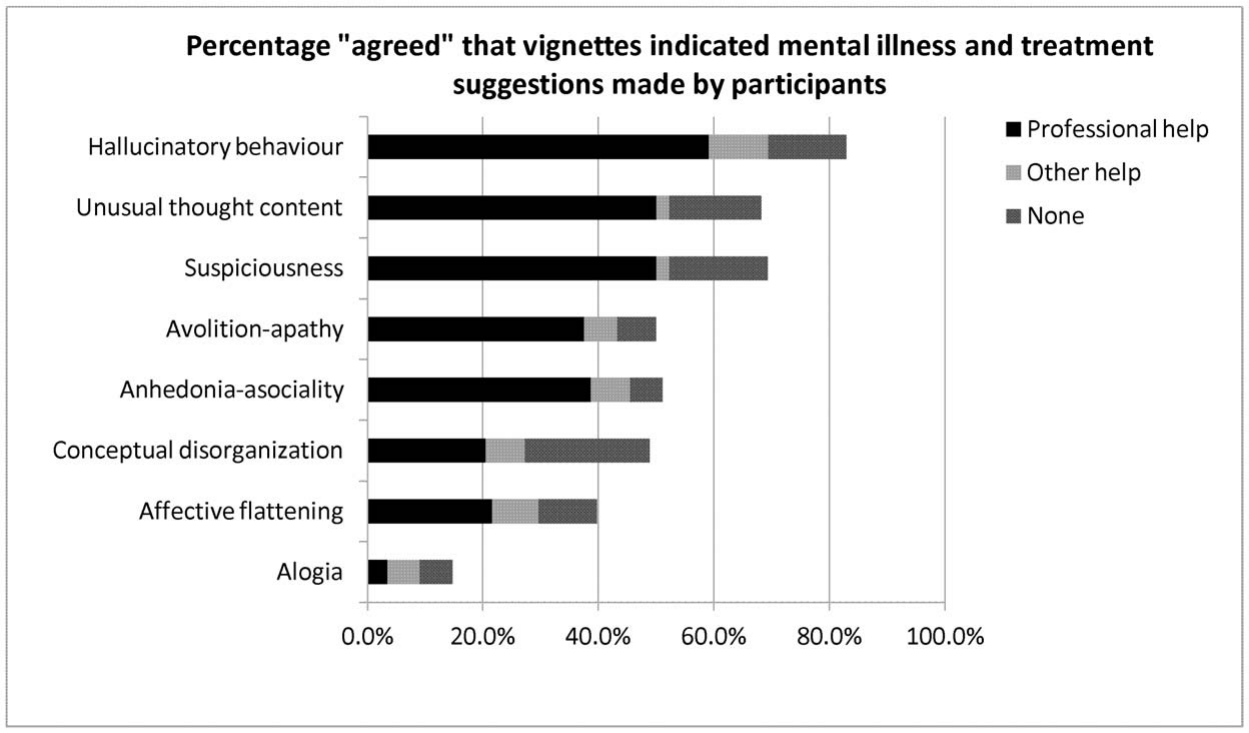
Treatment Suggestion - frequencies, percentages and chi-square analyses of treatment suggestions made by participants.

Figure 2 shows absolute percentage of the whole sample population for the three different help types (‘Professional help’, ‘other help’ or ‘don’t know’ for each symptom vignettes for those participants who had **initially agreed** that a given symptom vignette indicated mental illness, who also suggested help/treatment required.

These findings which are illustrated in the figure can be summarised as following:

1. For ‘hallucinatory behaviour’ (positive symptom), around 60% of the whole sample population recommended ‘professional help’ (see Figure 2 where 80% of the same participants suggested help/treatment required).
2. For ‘unusual thought content’ and ‘Suspiciousness’ (positive symptoms) around 50% of the whole sample population recommended ‘professional help’ (also see Figure 2 where 70% of the same participants suggested help/treatment required).
3. For ‘Avolition-apathy’ and ‘Anhedonia-asociality’ (negative symptoms) less than 40% of the whole sample population recommended ‘professional help’. (whereas in Figure 2, 50% of the same participants suggested help/treatment is required).
4. For ‘Conceptual disorganisation’ (positive symptom) and ‘Affective flattening’ (negative symptom), only 20% of the whole sample population recommended ‘professional help’ (whereas in Figure 2, 50% for ‘Conceptual disorganisation’ and 40% for ‘Affective flattening’ of the same participants suggested help/treatment is required).
5. For ‘Alogia’ (negative symptom), participants did not consider that the symptom vignette indicated mental illness in the first place, however, this is really negligible.
6. Other than ‘Professional help’ participants were often not sure which treatment that they could recommend.

In general, even those who originally agreed that the vignettes indicated mental illness and help/treatment is required, and ***suggested*** ‘Professional help’ constituted often 50% or even less than 50% of the sample population except for ‘Hallucinatory behaviour’ of just under 60%.

The results of Chi-square tests revealed that main effects were all significant (all at p<.05 -p<.001) except for ‘Alogia’ (χ_2_ = 0.62, p = 0.74).

Further post-hoc analyses revealed the following: For ‘Avolition-apathy’ (negative symptom), participants were more likely to suggest ‘professional help’ than alternatives forms of treatment (‘other’) (χ_2_ = 20.63, p<0.001) or for ‘don’t know’ (χ_2_ = 18.69, p<0.001). For ‘Anhedonia-asociality’ (negative symptom), a similar pattern was found (χ_2_ = 19.60, p<0.001; χ_2_ = 21.56, p<0.001, respectively). This was the same for ‘Hallucinatory behaviour’(positive symptom) (χ_2_ = 30.31, p<0.001; χ_2_ = 25.00, p<0.001, respectively), and for ‘Unusual thought content’ (positive symptom) (χ_2_ = 38.35, p<0.001; χ_2_ = 15.52, p<0.001, respectively), but participants were also more likely to suggest ‘don’t know’ than ‘other’ treatment (χ_2_ = 9.00, p<0.005). For ‘Suspiciousness’(positive symptom) this was also the case (χ_2_ = 38.35, p<0.001; χ_2_ = 14.25, p<0.001 and χ_2_ = 9.94, p<0.005, respectively), and for ‘Conceptual disorganisation’ (negative symptom)participants were more likely to suggest ‘professional’ treatment than ‘other’ (χ_2_ = 6.00, p<0.05), and to suggest ‘don’t know’ than ‘other’ treatment (χ_2_ = 6.76, p<0.01), but were no more likely to suggest ‘professional’ treatment than ‘don’t know’ (χ_2_ = 0.03, p = 0.87).

## Discussion

The results of the current study can be summarized as follows: (i) Participants were generally more likely to perceive positive symptoms of schizophrenia as indicative of mental illness than negative symptoms; (ii) Participants were more likely to suggest some form of help or treatment for positive symptoms than negative symptoms; and (iii) Participants were more likely to suggest professional help for positive symptoms than negative symptoms.

### Perception of Negative and Positive Symptoms of Schizophrenia

In general, the positive symptoms of schizophrenia such as ‘Hallucinatory behaviour’, ‘Unusual thought content’, ‘Suspiciousness’ and ‘Conceptual disorganization’ were perceived as being more indicative of mental illness than the negative symptoms of schizophrenia such as ‘Avolition-apathy’, ‘Alogia’, ‘Anhedonia-asociality’ and ‘Affective flattening’.

This was also reflected when participant responses were categorized into three groups: ‘Agree’, ‘not sure’ and ‘disagree’ as tabulated in Table 2. Participants were more likely to agree that positive symptom vignettes indicated mental illness than otherwise in all cases. However, for ‘Avolition-apathy’ and ‘Anhedonia-asociality’, the participants were also likely to agree that negative symptom vignettes indicated mental illness. It is possible that these two symptom vignettes were much more salient in their depiction of depression-like symptoms than ‘Affective flattening’ or ‘Alogia’. ‘Alogia’ (meaning ‘poverty of speech’) in particular was clearly not considered anything like a symptom of mental illness by the population sample. The validity of this particular vignette (as originally conceived by McEvoy et al. [30]) may be called into question, as it might be the case that the vignette has not captured the true sense of ‘Alogia’ or a schizophrenic symptom.

The findings of the current study that positive symptoms were perceived as more discernible signs of mental illness than negative symptoms by a lay population could add further understanding to previous research into treatment delays for schizophrenia**.** Addington et al. [16] had found that patients experiencing a first-episode psychosis were likely to seek help in the presence of overt positive symptoms, but not as likely to seek help when only experiencing depression-like symptoms and functional decline. It may generally be the case that the positive, psychotic symptoms of schizophrenia are more ‘recognisable’ or concerning to people than the negative, more depressive symptoms of schizophrenia.

This is in contrast to O’Callaghan et al.’s [9] suggestion that the recognisability of depressive symptoms (e.g. ‘avolition-apathy’, ‘anhedonia-asociality’, ‘affective flattening’ and ‘alogia’) may be prompting people who are developing schizophrenia to seek help. However, while it is still accurate to state that the depressive (negative) symptoms of schizophrenia may still be a concern to people, as was also suggested by Addington et al. [16], and has been observed in the current study (see Table 2, and Figure 1 ), it is still a troubling reality that help-seeking delays can be exceptionally long in the early, prodromal stage of illness when these symptoms are often present.

Indeed, our data suggested that people generally do not confidently recognise some negative symptoms as signs of mental illness and that most people do not necessarily consider professional healthcare a priority for these symptoms as illustrated in Figure 2. The current results are thus in agreement with Addington et al.’s [16] findings in first-episode psychosis patients. In addition, O’Callaghan et al. [9] found that only 40% of people who had developed psychotic (positive) symptoms sought help for themselves, and our data also showed that lay people may not recognise the importance of treatment or professional help for some psychotic symptoms such as ‘Unusual thought content’ (50% of the sample population shown in Figure 2), ‘suspiciousness’ (50% of the sample population shown in Figure 2) and ‘conceptual disorganisation’ (20% of the sample population shown in Figure 2). Such evidence suggests that treatment delays may be related to a general under-recognition of early symptoms, both positive and, in particular, negative symptoms, and the importance of early intervention for schizophrenia in the community. Similar suggestions were made by other researchers [8], [9], [10], [16], [22], [23], [33].

### Help-seeking Perceptions of Schizophrenia Symptoms & Treatment Recommendations for Schizophrenia Symptoms

As shown in Figure 1, participants were more likely to suggest professional help or treatment for positive symptoms, particularly ‘Hallucinatory behaviour’, ‘Unusual thought content’ and ‘Suspiciousness’ than any of the negative symptoms. In fact, ‘Alogia’ and ‘affective flattening’ received notably few recommendations for help or treatment. Interestingly, one positive symptom, i.e., ‘Conceptual disorganization’ received levels of help or treatment recommendation more similar to the negative symptoms. It is possible that, in isolation, the ‘strength’ of these vignettes for depicting mental illness was not enough for participants to consider help or treatment necessary. Participants may be considering symptoms such as ‘hallucinatory behaviour’ as ‘red flags’ for serious mental disorder and requiring attention, whereas for the aforementioned vignettes they may only be considered important in the context of a constellation of symptoms.

It is important to note, however, that most symptoms were (except for ‘Alogia’ and ‘Affective flattening’), in general, recognised as being at least somewhat indicative of mental illness by the participants in this study (see Table 2). It is the fact that lack of *awareness* of the need for professional health assessments or intervention (see Figure 2) was prominent that in a general sense there may be, as Jorm [25] and Lauber et al. [26] asserted, a lack of ‘mental health literacy’ in the general population, or about schizophrenia at least.

Indeed, the current study revealed that the terms ‘schizophrenia’, ‘psychosis’ or ‘Anti-psychotics’ were only mentioned on 19 occasions out of a total of 704 symptom vignette responses. This is despite the fact that participants were not directly asked for suggestions of causation. Lack of knowledge about schizophrenia has been observed in first-episode psychotic patients with long durations of untreated psychosis, including a study that found 74% of patients lacked knowledge about psychosis [34].

Interestingly, Joa et al. [33] found via the Early Treatment and Intervention for Psychosis (TIPS) Programme in Norway and Denmark between 1997 and 2000 that the use of intensive information campaigns (ICs), educating the general public about the early signs and symptoms of schizophrenia and the importance of early intervention, significantly reduced durations of untreated psychosis, and patients were less severely ill on intake compared to a control programme.

Indeed, the current findings strongly indicate that an improvement may be needed in educating the public about the early signs and symptoms of psychosis and schizophrenia, as advocated by a number of studies [9], [10], [16], [22], [33]. This study advocates the use of more public information campaigns in a vain similar to the TIPS programme [33], in the case of psychosis, or other major campaigns used for common medical problems e.g. NHS Act F.A.S.T stroke campaign. Such campaigns to improve ‘mental health literacy’ could educate the public in symptom detection and help-seeking for psychosis. Improving public awareness of schizophrenia in such a way may help reduce the long delays to treatment often found in people developing first-episode schizophrenia.

Joa et al. further pointed out that an awareness intervention programme in Australia conducted by Krstev et al. [35] was less successful. This was because Krestev et al. only targeted schools and primary healthcare professionals, and not the wider public. It is interesting that a large body of research is devoted to cutting down treatment delays for cancers, strokes and heart disease, and visible public awareness campaigns for symptom detection for these illnesses, yet there is much less known about interventions for schizophrenia and problems related to treatment delays [9]. Thus, by investigating the ‘mental health literacy’ of a lay population about schizophrenia (specifically in symptom recognition and help-seeking) and relating that to important clinical implications of the illness (e.g. Durations of Untreated Psychosis), this study has attempted to bring together two important areas of research in schizophrenia epidemiology. The findings of this study are ultimately supportive of efforts to improve the mental health literacy of schizophrenia in lay populations, which could in turn help reduce DUP.

### Further Implications

The current study was exploratory in nature, and for future research, a larger sample size as well as different ethnicities (e.g., British Asians, British Afro- Caribbean, British Chinese, and etc.) would be needed to be included to gain a better picture of the general public or different ethnic groups’ understanding of epidemiological factors relating to schizophrenia (e.g., Stone and Finlay [29]). Further, demographic information of participants, such as specific educational attainment details and information on any personal experiences of mental illness would also be needed to better understand how well the general public or different ethnic groups understand schizophrenia. Future studies should also consider other potentially important factors related to treatment delays, including the delays that occur in primary healthcare systems, which have been found to be an important variable [36].

### Conclusion

This study found that lay people are fairly adept at identifying some symptoms in particular positive symptoms of schizophrenia as indicating mental illness. However, negative symptoms appeared to be perceived as less discernible signs of mental illness than positive symptoms. Further, there appeared to be an ‘unawareness’ among the population that many of the symptoms may require professional assessment and intervention. The study also suggests a stronger need to educate the public about the early signs and symptoms of psychosis and schizophrenia.
